# The complete chloroplast genome of *Epimedium platypetalum* K. Mey. (Berberidaceae), a rare plant species from China

**DOI:** 10.1080/23802359.2021.1974968

**Published:** 2021-10-23

**Authors:** Xiang Liu, Yixin Zhang, Cheng Zhang, Chaoqun Xu, Weihan Qin, Guoan Shen, Baolin Guo

**Affiliations:** a Institute of Medicinal Plant Development, Chinese Academy of Medical Science, Peking Union Medical College, Beijing, China; b Chongqing Academy of Chinese Materia Medica, Chongqing, China

**Keywords:** Chloroplast genome, *Epimedium platypetalum*, infrageneric classification, phylogenetic analysis, Berberidaceae

## Abstract

*Epimedium* L. is an important medicinal herbaceous genus in the family Berberidaceae. *Epimedium platypetalum* K. Mey. is a plant species only narrowly distributed in the western part of China. Here, the complete chloroplast genome of *Epimedium platypetalum* was assembled. The chloroplast genome of *E. platypetalum* was 159,088 bp in length, with a total GC content of 38.79%. A total of 112 unique genes were identified, among which 78 are protein-coding genes, 30 tRNA genes, and four rRNA genes. Phylogenetic results revealed that *E. platypetalum* formed a sister relationship with *E. membranaceum* K. Mey. Our findings provided valuable data for future research on phylogenetic relationship and germplasm exploration within the genus *Epimedium*.

*Epimedium* L., the largest herbaceous genus of family Berberidaceae, contains over 60 plant species distributed very unevenly from North Africa (Algeria) to East Asia (Stearn 2002; Ying 2002). Since Linnaeus first identified the *Epimedium* species *E. alpinum* in 1753, controversies have existed all along in the infrageneric classification of*Epimedium* genus. Based on the C-banding of chromosomes, flower and leaf morphology, and geographical distribution, Stearn proposed a taxonomic classification system of *Epimedium* genus in 2002, in which *Epimedium* genus was classified into two subgenera (subgenus *Epimedium* and subgenus *Rhizophyllum*). The subgenus *Epimedium* was further divided into four sections (section *Epimedium*, section *Polyphyllum*, section *Macroceras*, and section *Diphyllum*). The largest section *Diphyllon* is exclusively comprised of more than 50 Chinese species, so it is believed that China should be the modern diversity center of *Epimedium* species (Stearn 2002; De Smet et al. 2012). The leaves of *Epimedium* plants have been used as a kidney-tonic and antirheumatic herb ‘Herba Epimedii’ in traditional Chinese medicine for more than 2000 years. Pharmacological studies have verified that Herba Epimedii has wide-reaching activities, including anti-tumor, anti-aging, regulating bone remodeling, improving immunological function, and so on (Ma et al. 2011; Fan and Quan 2012; Yang et al. 2019).

The taxonomic classification of genus *Epimedium* remains debatable since interspecific hybridization and gene introgression has resulted in an extreme complexity of interspecific relationship. Chloroplast genomes play an important role in phylogenetic research due to their special advantages of small size, mostly single-copied, highly conserved gene composition and genome structure, and relatively moderate nucleotide substitution rate (Nock et al. 2011; Zhang and Li 2011; Li et al. 2015). Therefore, it is essential for resolving the infrageneric phylogenetic relationship of *Epimedium* genus to sequence and analyze chloroplast genomes of more species.

*Epimedium platypetalum* K. Meyer 1922 is narrowly distributed in the western part of China (He 2014). In 1922, K. I. Meyer published *Epimedium platypetalum* using the type specimen collected by Hans Wolfgang Limpricht (German botanist) in the Wenchuan County (Sichuan province, China) in 1914 (Meyer 1922). According to the record of the species distribution (He 2014), *E. platypetalum* is only found to be located in the west of Sichuan province and the south of Shanxi province, China. Despite the *E. platypetalum* plants from the two regions resembled in the petal shape (spurless), which is unique among *Epimedium* species, great difference existed between the *E. platypetalum* population in Shanxi and Sichuan provinces: the *E. platypetalum* population from Shanxi are observed with three leaflets and 2–6 flowers, whereas the *E. platypetalum* population from Sichuan are observed to contain 3–5 leaflets and 6–14 flowers. In the previously published research, Guo et al. had recently reported the chloroplast genome of *E. platypetalum* (voucher no.: SXLP, GenBank accession number: MT560421) which was sampled from the Liping town of Nanzheng County, Shanxi province (Guo et al. 2021). However, our samples were collected from the type locality (Wenchuan County, Sichuan province, China), in which only Professor Mikinori Ogisu had rediscovered *E. platypetalum* in 1993 (Stearn 1938; Ogisu 1996). Since the classification of *E. platypetalum* from these two regions has remained debatable all along, it is necessary to report the chloroplast genome of *E. platypetalum* collected from the type locality (Wenchuan County, Sichuan province, China) to clarify the phylogenetic position of these two ecotypes of *E. platypetalum*.

In this study, *E. platypetalum* was sampled from Wenchuan County of Sichuan province, China (latitude 31.3614 and longitude 103.4971). The specimen and extracted DNA were deposited at Medicinal Plants Authentication Center, Institute of Medicinal Plant Development, Chinese Academy of Medical Science (http://www.implad.ac.cn/, collected by Xiang Liu, zysliux@163.com) under the voucher number Liu18038. The genomic DNA was extracted from the fresh leaves of *E. platypetalum* with the modified CTAB method (Doyle and Doyle 1987), and was then used to generate libraries with an average insert size of 300 bp using the VAHTSTM Universal DNA Library Pren Kit (ExCell Bio. Biological Technology Co., Ltd., Shanghai, China). Genome sequencing was performed with the Illumina Novaseq 6000 platform (Illumina Inc., San Diego, CA), and 150 bp paired-end reads were generated. The assembly of chloroplast genome was conducted using the GetOrganelle v1.5 program (Jin et al. 2018) with *E. acuminatum* (GenBank accession number: NC_029941) as reference. The annotation of chloroplast genome was conducted through the online program CPGAVAS2 (Shi et al. 2019) and assisted with manual correction and the annotated genomic sequence was deposited into GenBank with an accession number (MW483078).

The complete chloroplast genome of *E. platypetalum* (MW483078) was 159,088 bp in length, which was 136 bp shorter than the *E. platypetalum* from Shanxi (MT560421), including two inverted repeat regions (IR_A_ and IR_B_, 27,718 bp) separated by a large single copy region (LSC, 86,581 bp) and a small single copy region (SSC, 17,071 bp). The total GC content was 38.79%, with IR regions (43.02%) higher than that in LSC (37.29%) and SSC regions (32.76%). A total of 112 unique genes were identified from the chloroplast genome of *E. platypetalum*, including 78 protein-coding genes, 30 tRNA genes, and four rRNA genes. The intron–exon structure analysis indicated that a total of 18 genes have introns, among which *pet*B, *pet*D, *rpl*16, *rpl*2, *rpo*C1, *rps*16, *trn*A-UGC, *trn*G-UCC, *trn*I-GAU, *trn*K-UUU, *trn*L-UAA, *trn*V-UAC, *atp*F, *ndh*A, and *ndh*B had one intron, whereas *ycf*3, *rps*12, and *clp*P contained two introns.

For determination of phylogenetic position of *E. platypetalum* from Sichuan (MW483078), phylogenetic analysis was conducted using the complete chloroplast genome sequences of *E. platypetalum* from Shanxi (MT560421) and other 10 species from the NCBI GenBank database. MAFFT v7 (Katoh et al. 2019) was applied to generate sequence alignment. Especially, sequence alignment of the two *E. platypetalum* chloroplast genomes revealed 239 variable sites, among which 66 were detected in CDS regions. The maximum-likelihood (ML) tree was constructed using the RaxmlGUI v1.5b2 program (Silvestro and Michalak 2012) with 1000 bootstrap replicates. The Bayesian inference (BI) tree was constructed with MrBayes 3.2.7 (Ronquist and Huelsenbeck 2003). The Markov chain Monte Carlo (MCMC) algorithm was run for 1,000,000 generations, with one tree sampled every 1000 generation still convergence (the average standard deviation of split frequencies <0.01). The first 20% of trees were discarded as burn-in, and the remaining trees were used to build a 50% majority-rule consensus tree. *Vancouveria hexandra* (Hook.) C. Morren & Decne was selected as the outgroup (Figure 1). As a result, the ML and BI phylogenetic tree displayed identical topologies. In particular, after the node defining a clade of *Epimedium membranaceum* K. Mey., *Epimedium stellulatum* Stearn and *E. platypetalum*, the *E. platypetalum* from Sichuan (MW483078) was sister to *E. membranaceum* K. Mey., and the *E. platypetalum* from Shanxi (MT560421) formed a sister relationship with *E. stellulatum*, indicating the difference between the *E. platypetalum* plants from the two distribution regions. Therefore, our study provided valuable information for the understanding of *E. platypetalum* and future phylogenetic and evolutionary studies of *Epimedium* genus.

**Figure 1.**
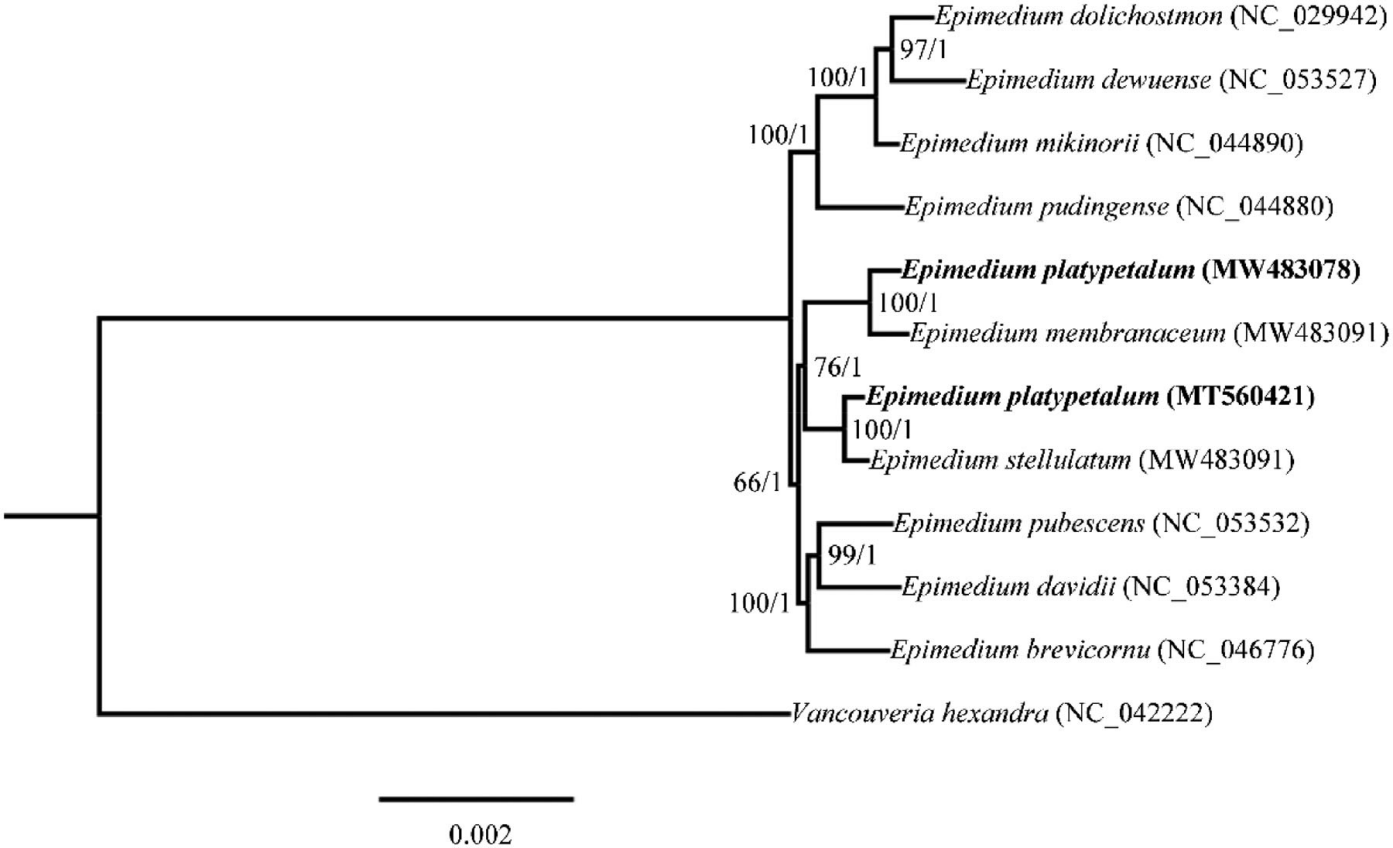
Maximum-likelihood (ML) and Bayesian inference (BI) phylogenetic tree based on complete chloroplast genomes of 11 species, with *Vancouveria hexandra* as outgroup. The support values at the nodes are for ML bootstrap support and BI posterior probabilities, respectively.

## Data Availability

The genome sequence data that support the findings of this study are openly available in GenBank of NCBI at https://www.ncbi.nlm.nih.gov/ under the accession no. MW483078. The associated numbers are PRJNA749730, SRR15254402, and SAMN20398933, respectively.
